# Natural History, Pathology, and Treatment of the Epidemic Fever at Present Prevailing in Edinburgh and Other Towns: Illustrated by Cases and Dissections

**Published:** 1844-07-01

**Authors:** 


### Natural History, Pathology, and Treatment of the Epidemic Fever, at present prevailing in Edinburgh and other Towns: illustrated by Cases and Dissections. By *John Rose Cormack*, M.D., F.R.S.E., &c. London, 1843.

In the number of the Edinburgh Medical and Surgical Journal, published on the 1st of January, 1844, we have a lengthened article from the pen of Professor Henderson, regarding the Epidemic prevailing in Edinburgh. The almost exclusive object of Doctor Henderson in that article seems to be to prove that the epidemic then prevailing, and which, for aught we know, still prevails in Edinburgh, is essentially distinguished from ordinary typhus by very striking and prominent characters. These distinguishing characters we shall not fail to notice in the course of our analysis of Doctor R. Cormack's monograph. The difficulties which encumber enquiries into the history and pathology of continued fevers, are universally felt and acknowledged. These difficulties may be referred to several sources, as, for instance, to the circumstance, that continued fevers have a number of characters common to them all. These coincidences, or points of resemblance between them, consisting of those prominent characters by which they are broadly distinguished from other diseases, the result has been, that their common disagreements with other diseases, and the similarity in certain points among themselves, have appeared to efface the features by which they should be distinguished from one another. Another source of difficulty is our not knowing the fundamental elements of their pathology; for, notwithstanding the reliance generally placed in nosological classification by symptoms, rather than by a reference to the conditions of the system on which these symptoms mainly depend, still our admission of actual and determinate distinctions among them is always strengthened by the discovery of pathological differences. Another source of these difficulties, which we shall notice, is our neglect of investigating carefully, and with exactness, the circumstances or causes by which fevers are produced. To be sure, as a general principle, the causes form not sufficient grounds either for making distinctions between diseases, or for grouping them together, as the same disease may acknowledge in different persons several different causes, and the same cause may occasion very different diseases; this latitude, however, existing between the causes of disease and their effects, does not exist between all causes and all diseases. There are some diseases which can only be produced by one cause, and certain causes which can produce but one kind of disease. There are many, very many examples, which corroborate this doctrine. Still the use which may be made of it in determining the distinctions among continued fevers is not very obvious—while it is granted that nothing is known of the actual nature of the poison or poisons by which continued fevers are produced, their production by some poison or other is still admitted. Some physicians, while they admit this, deny the specific nature of the poison which produces typhus fever, considering that its variability is one very influential source of the many diversities discovered among continued fevers, in which diversities they still recognize essentially the same disease. In our analysis we shall see how far Dr. R. Cormack is disposed to admit the distinctness of the present epidemic from the ordinary typhus.

Dr. Cormack divides his work into six chapters. The first treats of the *Moderately Congestive Form of the Disease.* In this form he first notices a peculiar *bronzed* appearance of the patient's countenance—the patient is first seized with coldness, rigors, headache, pain in the back, and prostration of strength. The cold terminates after a period varying from half an hour to several hours, when the headache generally becomes more severe—a dry burning heat comes out over the whole body, accompanied by thirst and general uneasiness. This hot stage is succeeded by a sweat, usually very profuse, continuing for a number of hours, seldom attended with relief to the headache. Sometimes the sweating is delayed to two or three days after the seizure—sometimes also there is no well-marked hot stage between the cold and sweating fits. During the whole course of the disease the perspiration was observed to have a characteristic disagreeable smell, and an acid re-action. During the three stages of the initiatory paroxysm the pulse is rapid, sometimes as high as 150, seldom below 90, and commonly ranging between 90 and 120. This great frequency of the pulse in the new epidemic at an early period of its existence has been noticed by Professor Henderson in the paper already alluded to, as forming one of the grand points of dissimilarity between the epidemic at present prevailing and ordinary typhus. This extreme frequency of the pulse in the epidemic, according to Professor H., seems to be an occurrence warranting no apprehension of a fatal issue, and no anticipation of any greater severity in the other symptoms, than in such cases as have a pulse 20 or 30 beats lower. For the first 48 hours the tongue, Dr. Cormack tells us, continues moist, exhibiting at the same time a white or brownish yellow fur. Afterwards the tongue becomes dry, and longitudinally streaked on the centre with brown, in which state it continues till the approach of the crisis, generally on the fifth day. During the first four days, some of the patients have occasional short rigors; most commonly, however, a state of dry ardent fever exists, with occasional sweatings. In many even of the mild cases nausea and vomiting usher in and attend the sufferings of the first days. Pain at the scrobiculus cordis generally accompanies these symptoms; it is frequently present without them. Severe muscular and articular pain is also observed. Pain in the abdomen on pressure, especially above the pubes and over the liver and spleen, are commonly observed. In consequence of the symptoms now described, the patients enjoy but little or no sleep. A remission is common on the third day; and, on the fifth day, the violence of the disorder manifestly appears to be expended; this state is generally ushered in by profuse sweat, epistaxis, or diarrhœa. After this the pulse, tongue, and skin, become quite natural, and the facial bronzing is less marked.

On or about the 14th or 15th day from the commencement the patient relapses; or, in other words, has a paroxysm of fever similar to that which began his first attack. This is noticed by Professor Henderson as another striking point of dissimilarity between the typhus and the epidemic fever. He expressly states, in fact, that the latter disease very rarely consists of but one attack of a continued febrile condition, and so characteristic of the disease is it to present a succession of attacks with apyrexial intervals, that the term relapse appears to him not less inappropriate to the repetition of the fever, than if applied to the paroxysms of an intermittent. The onset and progress of the second attack is attended sometimes by severer, sometimes by milder symptoms, than those of the first. It is in this the abortions generally occur. A third attack sometimes occurs—this is generally mild.

In Chap. II. the highly congestive form of the disease is described. One of the most common symptoms of this is yellowness of the conjunctivæ, and of the whole surface of the body, generally appearing between the third and seventh day—being most intense on the face, neck, chest, abdomen, and thighs. Associated with this yellowness, there are generally depression, delirium, dusky and often porter-coloured urine, melænic stools, and hæmorrhages from some of the mucous membranes. In the worst cases, black coffee-ground matter is ejected from the stomach and passed per anum. Sometimes the black vomit occurs without the yellowness. Enlarged liver and spleen, and tender and tympanitic abdomen, are usual symptoms in cases characterized by yellowness or extreme congestion. A deep persistent purple colour of the face, appearing before or immediately after the invasion of the disease, is a certain prognostic of danger, and is seldom absent in those destined to be yellow. With the exception of the purple countenance, the symptoms which usher in the congestive form of the disease differ little from those attending the disorder in its milder degree. In the severe cases there is generally a remission, but no intermission, about the seventh day.

In Chap. III. the *Pathology of the Disease* is given. The author commences with detailing the positive and negative characters, which distinguish the present epidemic from the fever which generally prevails in Edinburgh, viz.

1. *The sudden and violent invasion of the disease.*
2. *Bronzing, leadening, or purpling of the countenance before and after seizure.*
3. *The almost uniform occurrence of one or more relapses.*
4. *The unusual number of cases with yellow skin, black vomit, and hæmorrhage.*
5. *The short duration of the pyrexial state, and its mode of termination.*
6. *The severe muscular and articular pain.*
7. *The absence of the rosy, elliptical eruption, resembling measles in the present epidemic.*
8. *Severe vomiting is much more common, as are likewise gastric, gastro-hepatic, gastro-splenic, and gastro-enteric symptoms.*

With respect to the sudden and violent invasion of the disease, the present epidemic resembles that which prevailed here in 1817-20, and which was described by Dr. Welsh. The bronzing, leadening, and purpling of the countenance bear a strong resemblance, according to medical men familiar with the remittents and intermittents of Canada, the West Indies, and Italy, to what they have seen in persons affected with them in those countries. This phenomenon forms an interesting point of resemblance between the yellow fever of hot countries and our present epidemic. The recurrence of relapses forms another striking character of this epidemic, and forms also a point of contrast between it and typhus, in which such a recurrence is rare, and may generally be traced to errors in diet, or some other indiscretion. In the epidemic of 1817-20, relapses were also observed.

Yellowness of the skin and eyes, which are observed in the present epidemic, are also noticed by Dr. Welsh in the fever of 1817—20. Yellowness and black vomit have generally been taken as characters of special disease. Remittent and continued fevers, when prevailing epidemically, have been described under the name of yellow fever, a term applied to fevers differing essentially from one another in their character, constitution, and mode of propagation. This indefinite and vague use of the term yellow fever, however, should be banished from the scientific nomenclature of disease, as yellow skin, and black vomit, black stools and urine, and hæmorrhages, are consequences of congestion, which is liable to occur in fevers of all types and countries. Dr. Christison, in his work on Continued Fever, in the Library of Medicine, mentions "disorder of the hepatic system, accompanied with jaundice," as "an important, though rare complication." He says, that "it occurs chiefly in the Autumn months, and principally in those epidemics, where the inflammatory type is prevalent. Dr. Stokes and Dr. Graves describe some cases of yellow fever which they treated in the Meath Hospital of Dublin during the epidemic of 1827. On this occasion Dr. Graves made the remark that there is not so much difference between the diseases of Ireland and warmer countries as has been imagined; that they differ as to their degree, but not as to their pathology. Dr. Graves states, that in fifteen cases he could detect no difference in the symptoms which they presented, and those which characterize tropical yellow fevers. In both, he says, patients become yellow from absorption of bile into the system. Tenderness of the epigastrium and vomiting were present in both. The black vomit in the true yellow fever consists of a sanguineous fluid mixed with the vitiated secretion of the stomach and blood—this forms the coffee-grounds. According to the same authority, the seat of the disease is a violent inflammation of the mucous membrane of the stomach and duodenum. The last piece of pathology Dr. Cormack considers rather as an opinion than as an acknowledged fact; still the observation that the mucous membrane of this part of the intestinal canal was found dark purple, soft, and semi-fluid, Dr. C. considers valuable, as being the record of physical appearances actually seen. In the yellow cases of the fever now prevailing in Edinburgh, this same altered state of the gastro-intestinal mucous membrane is found; but there is no lymph effused—nothing, in fact, indicative of inflammation, unless in some severe cases which linger long.

Various modes have been offered to account for the yellowness of the skin in yellow fever. Some have ascribed it to general ecchymosis; and probably with justice in some instances; for Andral has shewn that it is this which produces the saffron colour of the skin in what is improperly called the jaundice of new-born children. This explanation, however, can only apply to those cases in which the yellowness is partial, or limited to particular parts of the body. The fluid in the cavities, as in the present epidemic, and the urine, have often a yellow tinge. Fordyce attributes the yellow skin to a redundant secretion of sebaceous matter. Dr. Cormack considers the most rational explanation to be, that it is the result either of an absorption of bile, or of its non-elimination from the blood.

With respect to *the short duration of the pyrexial state, and its mode of termination,* the author observes that the general occurrence of the crisis so early as the fifth day distinguishes this fever in a marked manner from typhus. The termination in the former is also much more commonly indicated by sweating, or other critical evacuation.

*The severe muscular and articular pains in the disease, and during convalescence,* form a rather interesting peculiarity of the present fever. The patients have acute rheumatic attacks, and occasionally acute pains in the feet, just like those which were affected with the epidemic of 1817-20. Dr. Cormack has found the arthritic and general pains in the jaundiced cases to be most severe; an observation worth recording, from the connexion which subsists between jaundice and rheumatism.

The absence of the rosy elliptical eruption resembling measles in almost every case in the present epidemic, constitutes one of the most remarkable distinctions between it and that which has been common in Edinburgh for a number of years past.

*Severe vomiting is much more common, as are likewise gastric, gastro-hepatic, gastro-splenic, and gastro-enteric symptoms.* Even in the mild cases, more or less pain of the epigastrium and vomiting, are general symptoms. In the mild cases, the matters vomited are generally the ingesta tinged with green, of various degrees of intensity. In the most malignant of the yellow cases there is sometimes a fine inky sediment in the vomit; at other times, the grounds are grumous, varying in colour from dark brown to black. This grumous matter is no doubt blood extravasated from the capillaries of the stomach, and chemically altered by the action of the acids of the stomach on it. The *gastro-enteric symptoms* are, in general, obviously referrible to the congested and irritated state of the mucous membrane of the stomach and bowels. Much of the pain complained of in the bowels arises from gaseous distention. A few other of the most important points in the pathology of the disease are next noticed; such as, 1st, the state of the blood; 2nd, the origin and mode of propagation; 3rd, the structural lesions caused by the fever. That the blood is really in a dissolved state, is evident, 1st, from its imperfect coagulation when drawn from the veins of patients; 2nd, from the ecchymosis universally observed to surround flea-bites or other slight injuries of the skin; 3rd, from the frequent occurrence of purpurous spots; 4th, from the hæmorrhages; and 5th, from the discoveries made by the microscope.

With respect to the *origin* of the prevailing epidemic, Dr. Cormack thinks that facts are not wanting to render it probable that the disease has been imported. With respect to the mode of propagation, there is sufficient evidence of its being contagious in the fact, that almost all the clerks and others exposed to the contagion have been attacked. Dr. Cormack is of opinion that the contagion of this fever is subject to the same law as that which regulates common typhus fever; viz. that the poison in which it orginates does not extend for more than three or four feet from the patient.

With respect to the *structural lesions caused by the fever,* they were found to consist in abundance or even excess of bile, and a pervious state of the biliary ducts, and more or less congestion of organs, with frequently extravasation of blood in various situations.

In the *Fourth Chapter* are detailed the principal Sequelæ of the Fever. These are thus summed up :—1. *Ophthalmitis usually preceded by amaurotic symptoms.* 2. *Glandular swellings.* 3. *Boils and cutaneous eruptions.* 4. *Effusion into the knee-joint.* 5. *Swelled legs and ankles.* 6. *Pain in the feet, with and without swelling.* 7. *Paralysis of the deltoid and other muscles.* 8. *Sloughing of parts.*

In the *Treatment* of this, as of every species of fever, the only general rule, which can be safely followed is that so correctly laid down by Cullen, and so urgently insisted on by Dr. Alison, viz. "*to obviate the tendency to death;*" with respect to the present epidemic there are, according to Dr. Cormack, three states most apt, either separately or conjointly, to cause death, and which, therefore, ought to be anxiously looked for, and, if possible, promptly corrected. They are—

1st. Congestion of the mucous membrane of the stomach and intestines, terminating in effusion of blood and subsequent destruction of large portions of this tissue.

2nd. Congestion of one or more of the abdominal viscera, particularly of the liver and kidneys, disabling them from the performance of their secretive functions and thereby rendering the blood impure.

3rd. Debility and sinking.

The best means to prevent these evils are the cautious but steady use of purgatives, the determining of blood to the surface and extremities, and in some cases its abstraction. When the kidneys are not duly performing their functions, a small bleeding from the lumbar region by cupping, or even a dry cupping, in those in whom depletion would be hazardous, proves of great benefit. When there is debility and sinking, cordials must be administered ; and, if this be combined with nausea or vomiting, they must be combined with sedatives.

With respect to the abstraction of blood, Dr. Cormack mentions that few of his patients bore the loss of more than ten ounces, and several became affected with vertigo and faintness, after two or three only had flowed. Some who, whilst the blood was flowing, declared themselves quite relieved from the head-ache, within half an hour after the arm was bound up were found to be suffering just as much as ever. He found that the most severe headaches were always far more effectually and uniformly relieved by the combined operation of a purgative, a pediluvium, and the constant application of cold to the head and the back of the neck. In several cases of pulmonary inflammation, Dr. Cormack found all the symptoms disappear under the use of antimony and morphia combined; or the liberal use of morphia and ipecacuan lozenges. These remedies, when used along with fomentations, sinapisms, or blisters, are safe, and generally efficient substitutes for local bleeding, in thoracic complications.

In a word, he conceives that the cerebral, pulmonary, and abdominal complications, in which it is proper to abstract blood, are extremely rare, and that in very many instances it is a most hazardous practice. In some of the more severe cases of abdominal pain, and extreme tenderness on pressure, the patients have been well brought through simply by the diligent use of copious warm poultices and fomentations. Enlarged and tender spleens have often done well with this simple treatment.

With respect to the application of cold to the surface, cold water to the head was, in general, found to be quite sufficient to allay the headache. In some instances, muriate of ammonia and other evaporating lotions were used. The aspersion of the arms and chest with cold water had the effect of calming several restless and irritable patients.

Medicines which act on the kidneys, when the urine was very scanty and scalding, and pain complained of in the loins, have proved serviceable —those used were the nitrate of potash, spirit of nitrous æther, and other diuretics. Warm fomentations to the loins, cupping, &c. act well in exciting the kidneys to renewed secretion, probably by preventing or moderating the congestion of these organs. Whenever alarming head symptoms occurred, associated with suppression of urine, he always directed attention to the kidneys.

We must here close our analysis of Dr. Cormack's monograph, in which will be found many and valuable practical remarks.

